# Supplementary material for: Room temperature 3D carbon microprinting
Source: Nat Commun. 2024 Mar 29;15:2745. doi: 10.1038/s41467-024-47076-z (PMC10980711; doi:10.1038/s41467-024-47076-z)
Supplement: Supplementary file 1 — Supplementary Information [file 41467_2024_47076_MOESM1_ESM.pdf]

## Supplementary Information for

# Room temperature 3D carbon microprinting

Fernand E. Torres-Davila[1,2], Katerina L. Chagoya[3], Emma E. Blanco[4], Saqib Shahzad,[5] Lorianne R. Shultz-Johnson[4], Mirra Mogensen[1,4], Andre Gesquiere[1,4], Titel Jurca[1,4,6], Nabil Rochdi[7,8], Richard G. Blair[6,9,*], Laurene Tetard[1,2,*]

[1] NanoScience Technology Center, University of Central Florida, Orlando, FL, USA.

[2] Department of Physics, University of Central Florida, Orlando, FL, USA.

[3] Department of Mechanical and Aerospace Engineering, University of Central Florida, Orlando, FL, USA.

[4] Department of Chemistry, University of Central Florida, Orlando, FL, USA.

[5] Department of Materials Science and Engineering, University of Central Florida, Orlando, FL, USA.

[6] Renewable Energy and Chemical Transformations (REACT) Cluster, University of Central Florida, Orlando, FL, USA.

[7] Laboratory of Innovative Materials, Energy and Sustainable Development (IMED-Lab), Cadi Ayyad University, Marrakesh, Morocco.

[8] Department of Physics, Faculty of Sciences Semlalia, Cadi Ayyad University, Marrakesh, Morocco.

[9] Florida Space Institute, University of Central Florida, Orlando, FL, USA.

*Corresponding authors. Email: Richard.Blair@ucf.edu and Laurene.Tetard@ucf.edu

### Defect engineering in *h*-BN and reaction

*h*-BN was milled to introduce a similar concentration of defects as described in the methods and in previous work by Ding et al.[1] We optimized milling conditions (milling time, number, and size of spherical milling media) by evaluating the percentage mass uptake of hydrocarbon after pressurization of 2 g of the milled powder with hydrocarbon for 24 h. Milling time of 20 to 120 min with a single ball of 0.75 in (~19 mm) led to the highest hydrocarbon uptake, as shown in Supplementary Fig. 1.

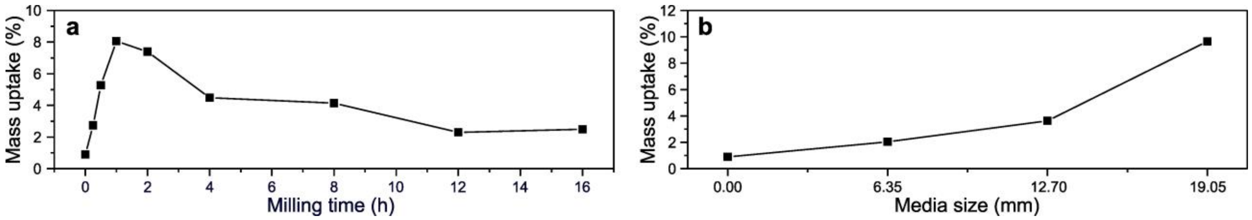


**Supplementary Fig. 1: Analysis of hydrocarbon mass uptake for *dh*-BN obtained by milling using spherical zirconia milling media. a** Propene mass uptake as a function of milling time. **b** Propene mass uptake as a function of media size. Source data are provided as a Source Data file.

Substrates coated with the catalyst were placed in a custom reactor, as shown in Supplementary Fig. 2.

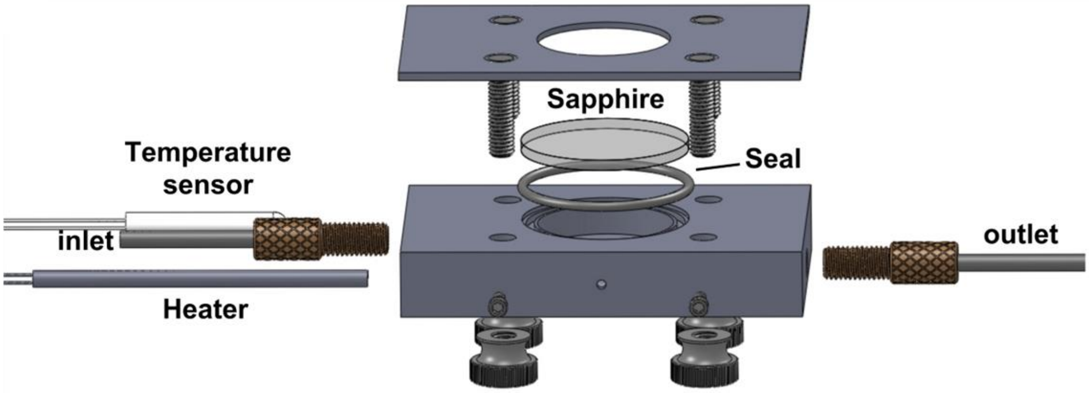


**Supplementary Fig. 2: Photoreactor designed to pressurize *dh*-BN with hydrocarbons for carbon growth.**

Illumination of *dh*-BN with visible light at 532 nm and power as low as 10 mW, focused on the surface of the powder with a 10× objective, resulted in a reaction at the surface

of the catalyst, leading to a color change from white to black, as shown in Supplementary Fig. 3.

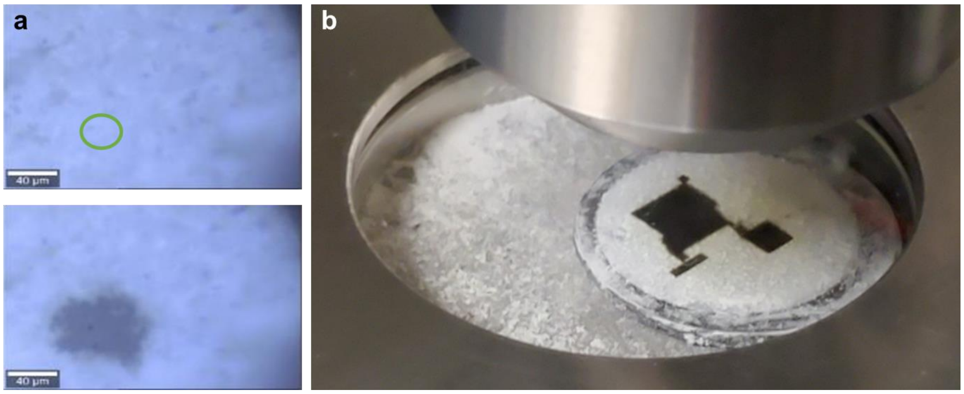


**Supplementary Fig. 3: Photographs of the reaction and carbon structures micropatterning on the *dh*-BN photocatalyst. a** *dh*-BN pressurized with the hydrocarbon before (top) and after (bottom) illumination with the 532 nm laser, at the position represented by the green circle in the top image. **b** Photograph of the patterned arrays obtained with different parameters.

Inspection of the reacted regions and the carbon rods (including the roots of rods) revealed some important features. For instance, the region consisting of the roots and region between the rods revealed a carbon-rich matrix with encrusted *h*-BN clusters, as shown in Supplementary Fig. 4.

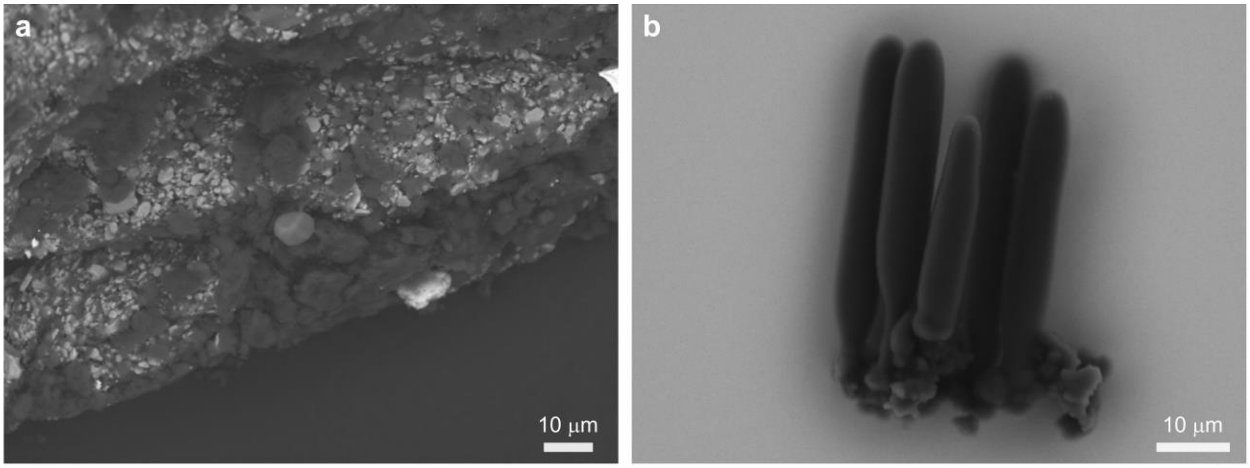


**Supplementary Fig. 4: SEM images of extracted carbon rods and underlying environment of the rod roots. a** SEM image of the region surrounding the roots of the rods recorded with backscattered electrons to highlight variations in chemical composition. The small flakes appearing with brighter contrast correspond to *dh*-BN flakes, inside the carbon matrix. **b** SEM image of a group of carbon rods extracted from the patterned region showing small residues of the roots where growth was initiated.

## Origin of the photocatalytic process for carbon synthesis

We evaluated the effect of light and temperature on the carbon microprinting process. Thermal catalysis at 500 °C with the same pressure of hydrocarbon gas adsorbed on *dh*-BN prepared in the same conditions did not result in any carbon formation on the catalyst. In addition, the effect of laser illumination on the gaseous environment was considered by depositing a discontinuous layer of *dh*-BN on a substrate and printing an array of carbon structures on an area encompassing regions covered with *dh*-BN and regions of bare substrate. Carbon microrods were formed only on regions covered with *dh*-BN, as shown in Supplementary Fig. 5, confirming that the process described here does not originate from direct decomposition of the reactant molecules by the laser illumination alone, but arises from *dh*-BN-assisted photocatalytic dehydrogenation.

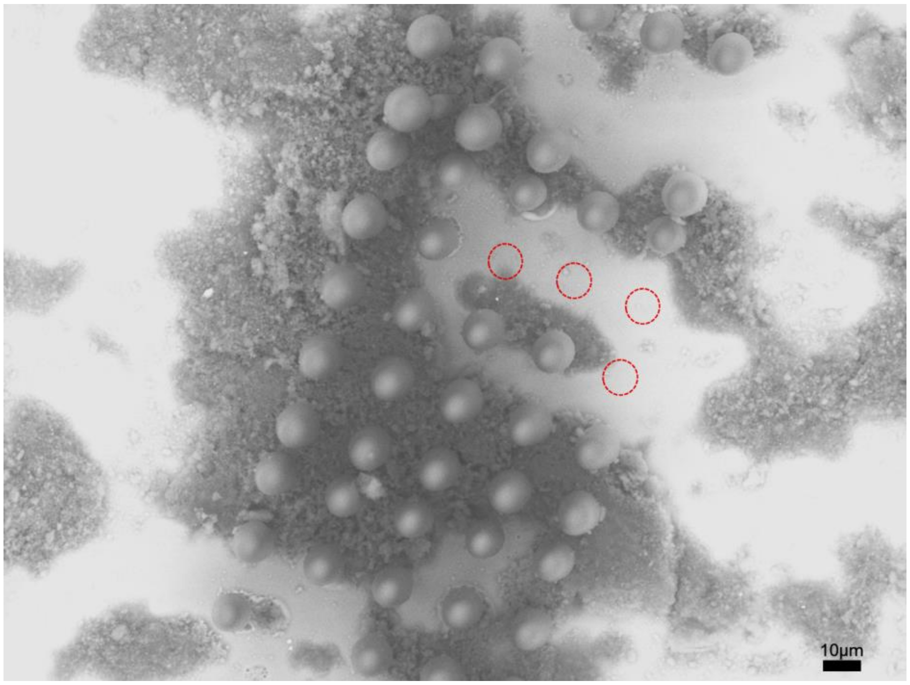


**Supplementary Fig. 5: SEM image of an array of carbon rod produced on a discontinuous layer of *dh*-BN.** The red marks indicate positions devoid of *dh*-BN where the laser illumination occurred with no carbon structures observed.

Next, we considered different light sources for the 3D microprinting process. Carbon microrods were obtained with laser sources emitting at 532 nm, 473 nm, and 405 nm, corresponding to photon energies above 2.33 eV. No carbon formation was observed with laser emitting at 633, 785 or 1064 nm (*i.e.,* for photon energies below 2 eV). The UV-Visible spectrum of *dh*-BN (Supplementary Fig. 6) supports this observation, as the absorption of *dh*-BN in the visible range is significantly higher than that of *h*-BN.

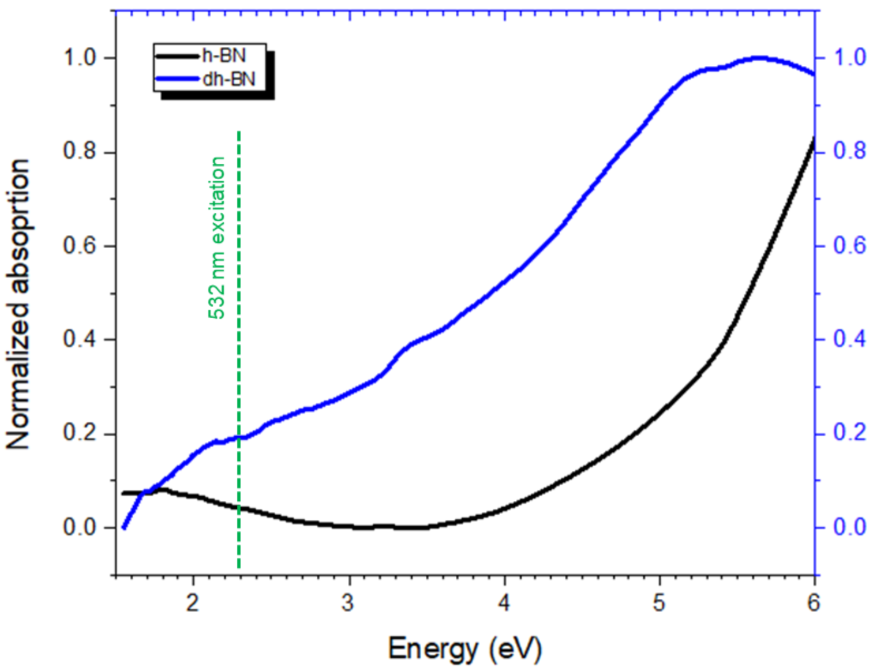


**Supplementary Fig. 6: UV-Visible absorption spectrum of *h*-BN before and after milling.** Milling creates defects, which correspond to new states in the band-gap of pristine *h*-BN that enable absorption of visible light and subsequently lead to the carbon growth presented here. Source data are provided as a Source Data file.

## Characterization of the carbon microstructures

### Analysis of the carbon growth by Raman spectroscopy:

Raman spectra of the carbon microstructures were analyzed following previously reported deconvolution procedures by fitting the G band as well as the D1, D3, and D4 bands.[2] A Lorentzian line shape centered at ~1590 cm$^{-1}$ was used for the G band (representing the $E_{2g}$ mode of graphitic-like structure). Lorentzian line shapes were used for the D1 and D4 modes and a Gaussian line shape was used for the D3 modes, overall describing the D ("defect") band centered around 1355 cm$^{-1}$, indicative of defects in the $sp^2$ aromatic network for carbon structures (Supplementary Table 1). The D2 band is generally centered at ~1620 cm$^{-1}$ and also indicates disordered graphitic lattice but relative to the $E_{2g}$ mode, indicative of surface graphene layers. This suggests that the crystallites are likely too small to observe this type of structural disorder. The lateral dimensions of the graphitic crystalline domains ($L_a$), the percentage of amorphous domains, and the level of hydrogenated carbon were estimated using the Knight and White's formula $L_a$ = 4.4 $A_G/A_{D1}$, following previous work discussed by Je Seong *et al.* [3] Further analysis of the G' Raman band is presented in Supplementary Fig. 7. The fit of this band with a single Lorentzian function suggests that the carbon material is turbostratic.

Next, Raman spectroscopy was used to analyze the evolution of the region illuminated with the laser as the reaction progressed. Supplementary Fig. 8 provides an overview of the changes in Raman fingerprints over the course of the reaction. The photoluminescence of the region undergoing the reaction leading to carbon growth was also recorded when using a 405 nm excitation (Supplementary Fig. 9) to display the emission peak at ~550 nm.

**Supplementary Table 1: Raman bands used for curve fitting of the Raman spectra collected on the reacted region of illuminated hydrocarbon-exposed *dh*-BN.**

| Band | Initial position [rel. $cm^{-1}$] | Line shape | Vibrational mode |
|---|---|---|---|
| G | 1580 | Lorentzian | $E_{2g}$ mode, indicative of the ordered graphitic lattice vibration[2,4,5] |
| D1 | 1335 | Lorentzian | $A_{1g}$ mode, indicative of disordered graphitic lattice (layer edges)[6-9] |
| D2 | 1620 | - | $E_{2g}$ mode, accounting for structural disorder in graphitic lattice (surface layers), indicative of surface to volume ration of graphitic domains[4,7,10,11] |
| D3 | 1500 | Gaussian | Indicative of amorphous carbon[11,12] |
| D4 | 1220 | Lorentzian | $A_{1g}$ mode, indicative of disordered graphitic lattice (polyenes, impurities) |

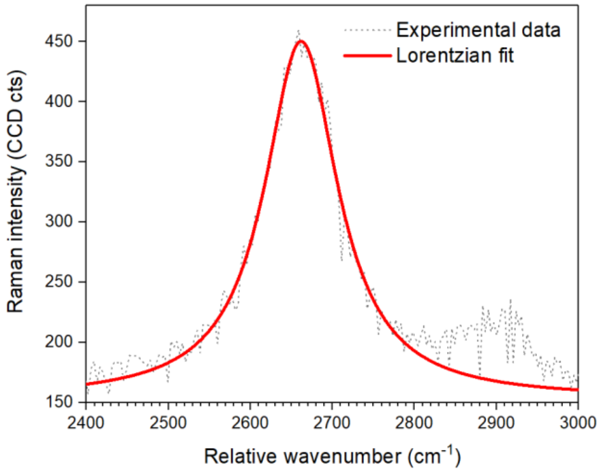


**Supplementary Fig. 7: G’ Raman band of the core of the carbon structure.** The G’ band can be fitted with a single Lorentzian function, indicative of a turbostratic carbon structure. Source data are provided as a Source Data file.

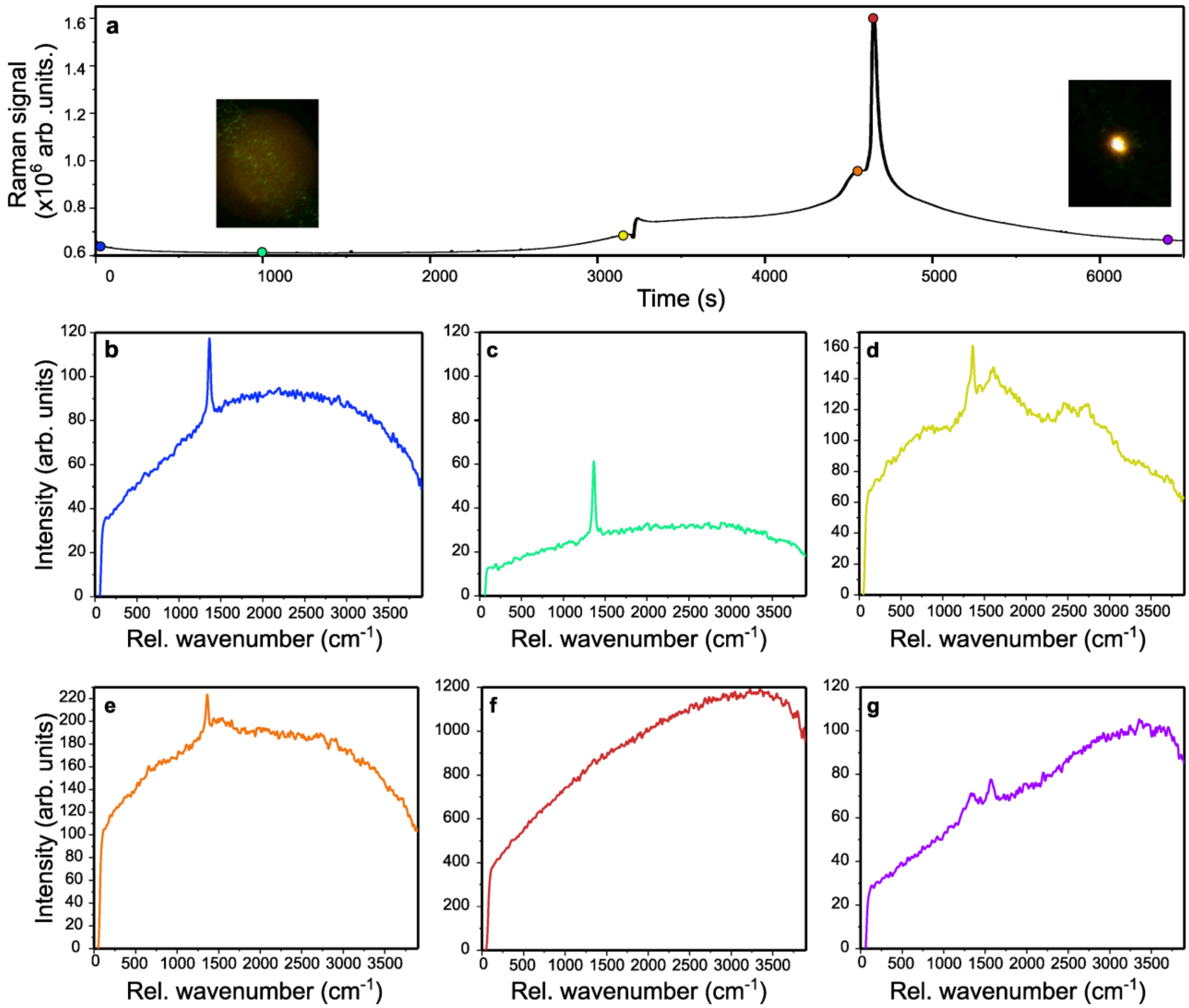


**Supplementary Fig. 8: Raman spectroscopy monitoring of carbon microrod growth as a function of illumination time. a** Time-evolution of the Raman signal calculated from the integral, over the relative wavenumber range 0–4000 $cm^{-1}$, of Raman spectra collected every 100 ms upon illumination of *dh*-BN. **b–g** Selection of Raman spectra collected before illumination (**b**) and after 1000 s (**c**), 3170 s (**d**), 4550 s (**e**), 4650 s (**f**), and 6330 s (**g**) of illumination. The spectra depict the evolution of the states formed on the catalyst leading to the carbon microrod growth. Source data are provided as a Source Data file.

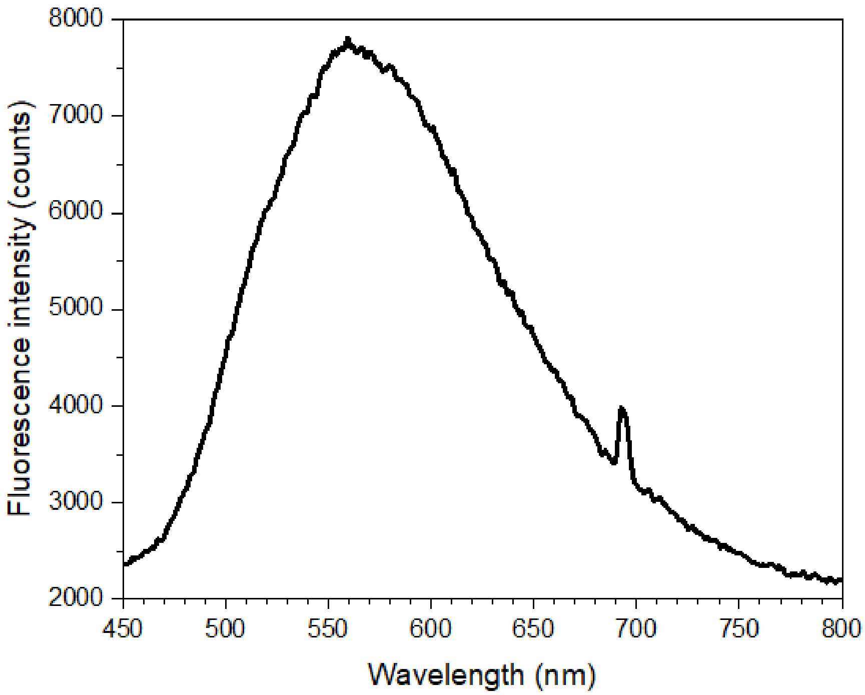


**Supplementary Fig. 9: Photoluminescence of the carbon growth using a 405 nm excitation laser.** The sharp band at 695 nm is attributed to the emission from $Cr^{3+}$ impurities in the sapphire window of the reactor. Source data are provided as a Source Data file.

## XPS Analysis of carbon microstructures

We examined the chemical makeup of the surface and immediate surface of unprocessed *dh*-BN and microrod tips, as shown in Supplementary Fig. 10. The unprocessed *dh*-BN powder was deposited on the quartz substrate in a microrod-free region, while the 1.2 mm-length carbon microrod arrays were patterned on *dh*-BN powder.

The survey spectrum of unprocessed powder (Supplementary Fig. 10c) shows the photoelectron lines for B 1*s* (~189.4 eV), N 1*s* (~398 eV), C 1*s* (~284.5 eV), O 1*s* and 2*s* (~531 and 23 eV, respectively), Si 2*s* and 2*p* (~151 and 99 eV, respectively), as well as the Auger KLL transitions of carbon (~1223 eV) and oxygen (~999 and 978 eV) [13]. Supplementary Fig. 11 displays the corresponding HR XPS spectra for *dh*-BN (bottom spectra). The B 1*s* and N 1*s* spectra highlight the signature of unaltered B–N bonds in *h*-BN at 190.4 eV (B–$N_3$ sites) and 398.1 eV (N–$B_3$ sites), respectively [14-17]. Additionally, the B 1*s* core-level scan shows a feature at 192.4 eV ascribed to B–O bonds that form upon exposure to air via oxygen bonding to milling-induced nitrogen vacancies [16,18]. On the other hand, the N 1*s* spectrum shows a feature at 400 eV, which is consistent with the formation of N–C bonds [18-20]. These results highlight the behavior of *dh*-BN defects when exposed to air. N defect sites show affinity with carbon while B sites exhibit affinity with oxygen [21]. With peaks relative to C–C (284.75 eV), C–O (286.3 eV), and C=O (288.2 eV) bonds, the C 1*s* spectrum mainly describes the chemical environments in adventitious carbon (*i.e.*, carbon species adsorbed on the quartz substrate). Similarly, the O 1*s* spectrum has a prominent feature at 532.7 eV that corresponds to O–Si bonds (quartz substrate) and a weak peak at 531.4 eV related to O=C bonds (adventitious carbon).

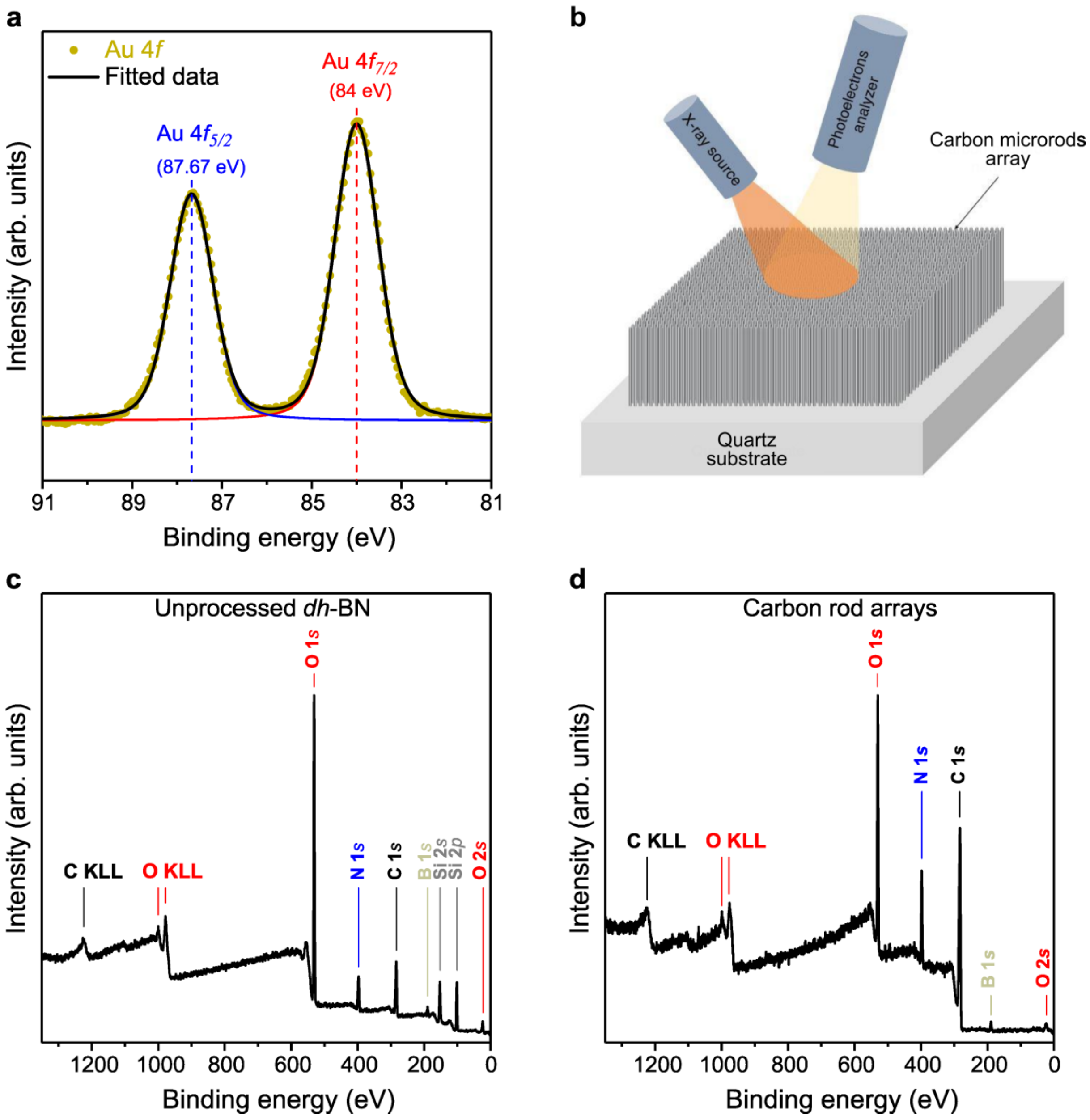


**Supplementary Fig. 10: XPS setup and survey analysis of unprocessed *dh*-BN and the carbon microstructure tips. a** Au 4*f* HR scan (symbols) from a gold specimen used to calibrate the HR XPS spectra. The Au 4*f* spectrum is fitted with the sum (black line) of two Doniach-Sunjic curves (red and blue lines) at 84 and 87.67 eV for the Au $4f_{7/2}$ and $4f_{5/2}$ lines, respectively. **b** Schematic of the XPS characterization method of the tips of 1.2 mm-length microrod arrays patterned on *dh*-BN powder placed in a square hole machined in a quartz substrate. The same method was used to analyze unprocessed *dh*-BN powder placed on a microrod-free region of the quartz substrate. **c**, **d** Corresponding XPS survey spectra of the unprocessed *dh*-BN (**c**) and the microrod tips (**d**). Source data are provided as a Source Data file.

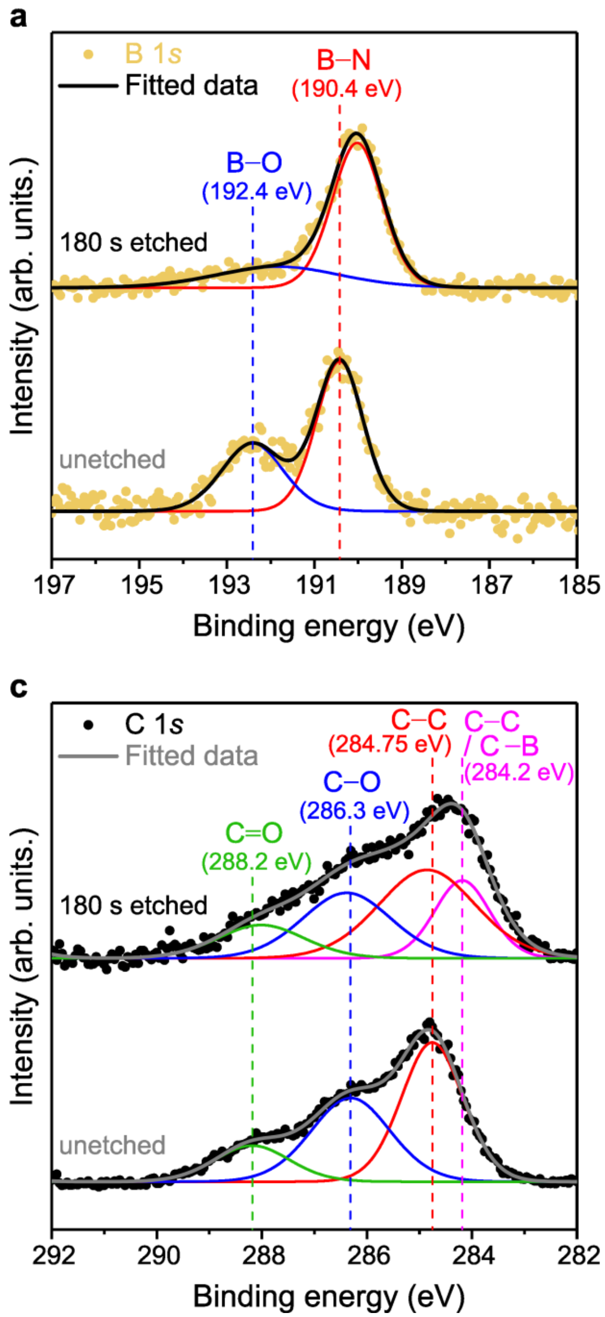


**Supplementary Fig. 11: HR XPS analysis of unprocessed *dh*-BN powder on the quartz substrate. a–d** XPS narrow scans (symbols) of B 1*s* (**a**), N 1*s* (**b**), C 1*s* (**c**), and O 1*s* (**d**) lines for unprocessed *dh*-BN powder placed on the quartz substrate. The spectra are fitted with sums (black/gray lines) of prominent Gaussian-Lorentzian curves (colored lines). The O 1*s* and C 1*s* spectra correspond mainly to bulk and adsorbed oxygen species from the quartz substrate and adventitious carbon, respectively. Source data are provided as a Source Data file.

The survey spectrum recorded on the carbon microrod tips (Supplementary Fig. 10d) highlights a carbon-rich and boron-deficient composition with estimated atomic contents of B, N, C, and O about 4%, 11%, 66%, and 19%, respectively. The corresponding chemical environments were examined using the HR XPS scans, shown in Supplementary Fig. 12 (bottom spectra). The B 1*s* spectrum consists of two distinct features at 190.2 and 191.85 eV, which are assigned to B–N/C and B–O bonds, respectively [20,22-24]. The N 1*s* spectrum exhibits two major peaks at 397.85 and 399.75 eV, which are consistent with nitrogen atoms bonded to boron (N–$B_3$ and N–$B_2$ sites) and to carbon, respectively. Accordingly, the C 1*s* narrow scan shows a major peak at 284.5 eV corresponding to C–C bonds, two peaks at 286.45 and 288.55 eV relative to oxidized carbon environments (C–O and C=O bonds), and two additional peaks at 283.45 and 287.65 eV. The peak at 283.45 eV corresponds mainly to C–C bonds in pentagonal defect sites (five-member rings) and to a smaller contribution from C–B bonds, while the peak at 287.65 eV is related to carbon bonded to nitrogen in pyrrolic or pyridone groups [25,26]. It is worth noting that the C–C feature (284.5 eV) is relatively broad, with a full width at half maximum (FWHM) of roughly 1.65 eV, which spans the chemical environments of $sp^2$ and $sp^3$ hybridized carbon [27]. Finally, the O 1*s* core level is composed of two main peaks that correspond to O=C (531.75 eV) and O–C (533.1 eV) bonds.

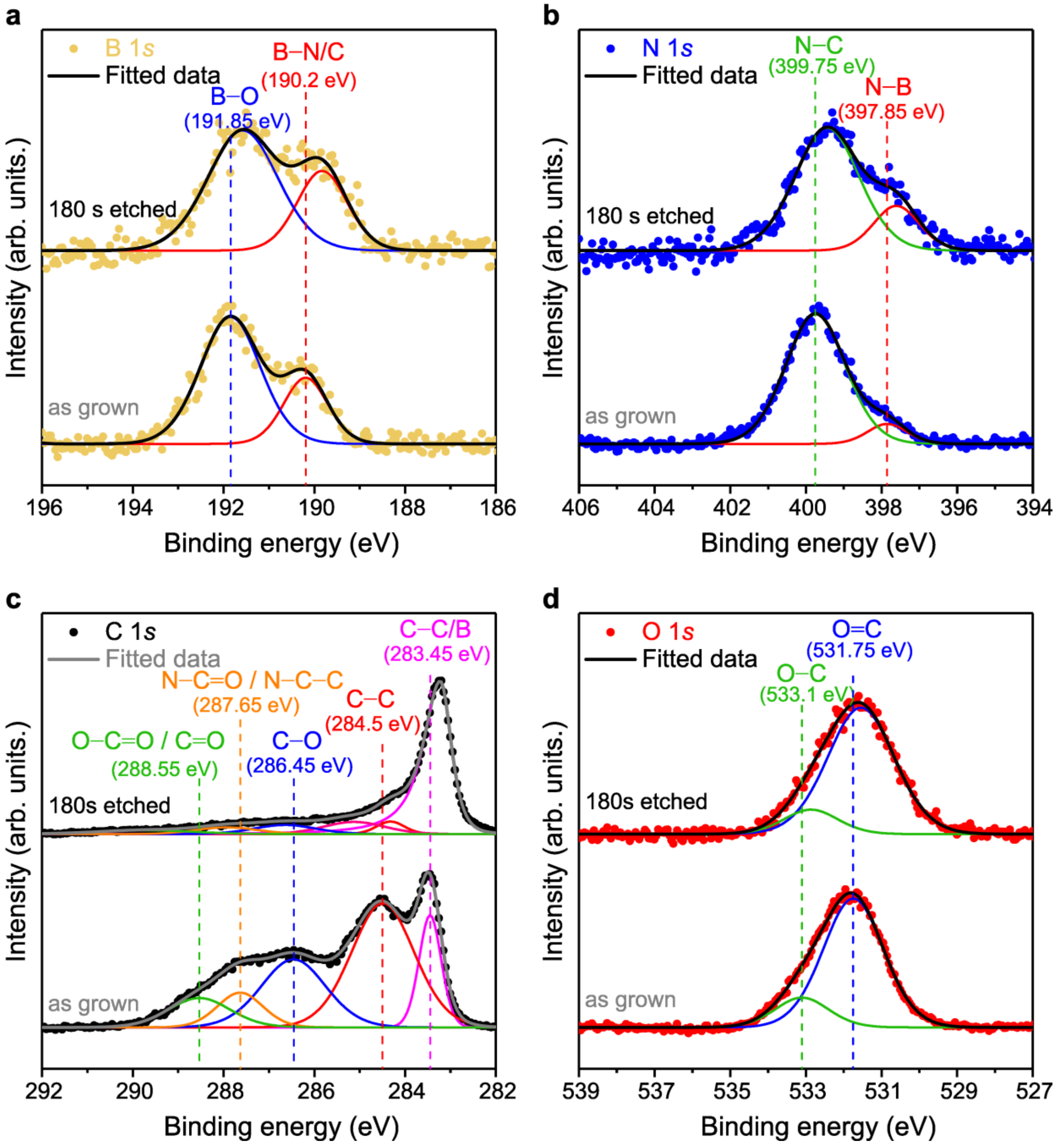


**Supplementary Fig. 12: HR XPS analysis of the carbon microstructure tips. a–d** XPS narrow scans (symbols) of B 1*s* (**a**), N 1*s* (**b**), C 1*s* (**c**), and O 1*s* (**d**) lines recorded on the tips of the as-grown (bottom spectra) and argon-etched (upper spectra) microrod arrays. The spectra are fitted with sums (black/gray lines) of prominent Gaussian-Lorentzian curves (colored lines). The C 1*s* spectrum for the etched microrod tips is fitted with the sum of an asymmetric Gaussian-convoluted Lorentzian line shape for the low-binding energy peak (magenta line) and symmetric Gaussian-Lorentzian functions for the other components. Source data are provided as a Source Data file.

The analysis of the C KLL Auger peak (Supplementary Fig. 13a) further supports the structure at the microrod tips since the estimated D-parameter, at approximately 12.4 eV (Supplementary Fig. 13b), is closer to $sp^3$-hybridized carbon than to $sp^2$ carbon (of about 13.2 and 23.1 eV, respectively) [28,29]. In addition, the valence band spectrum (Supplementary Fig. 13c) recorded on the microrod tips using Al $K\alpha$ excitation (1486.6 eV) shows a weak $p$-$\pi$ band at 2.9 eV, which evidences the $\pi$-bonding in $sp^2$ carbon [30]. The $sp^3$ hybridization is highlighted by the $s$-$\sigma$ band at 14.75 eV and, to a lesser extent, by the valence peak at 7.65 eV, which probably stems from the overlapping of the $sp^3$ carbon $p$-$\sigma$ and O 2p bands [30,31]. The broad feature at about 23.85 eV corresponds to O 2*s* states [13]. The valence band maximum was found to be 0.82 eV below the Fermi level as extrapolated from the leading edge of the valence peak (2.9 eV), as shown in Supplementary Fig. 13d.

We examined the composition of the microrod tips after an *in-situ* etching performed with a 0.6 mm-width argon ion beam at 4 keV for 3 min, as shown in Supplementary Fig. 12 (upper spectra). The resulting HR B 1*s* and N 1*s* spectra outline a relative increase in intensity of the B–C peak along with a decrease in the N–C peak intensity (Supplementary Figs. 12a,b). This suggests that the microrods' region exposed below the tip's surface, closer to the core, consists of a nitrogen-deficient CBN compound, presumably a defect-laden CBN material. This agrees with the downshift of the XPS peaks after the etching step, probably due to the ionic bombardment-induced decrease in the ligancy of atoms (*i.e.*, a decrease in the number of coordinated sites). These observations are strongly substantiated by the C 1*s* HR spectrum of etched microrod tips, which shows a major peak located at about 283.2 eV and smaller contributions at higher binding energies (Supplementary Fig. 12c). Thus, the ionic etching removes the C–C and C–N bonds, indicating that such carbon surroundings are present at the surface of the microrod tips. Interestingly, the resulting C 1*s* spectrum exhibits a graphite-like shape with a tail toward high binding energies, which is characteristic of the $sp^2$ hybridization. This strong asymmetry makes the curve fitting inaccurate when using Gaussian-Lorentzian functions exclusively. Indeed, the C 1*s* spectrum can be fitted using a Gaussian-convoluted asymmetric Lorentzian line shape centered at 283.25 eV and Gaussian-Lorentzian functions for the other components. The major

asymmetric peak is consistent with carbon bonded to boron (graphitic C–B bonds) [18,20,32,33], with a possible contribution from the above-identified defected cyclopentagonal carbon. Finally, the O 1*s* narrow scan reveals no substantial difference in composition after etching (*i.e.*, the O–C and O=C bonds), though there is a decrease in intensity of the corresponding peaks because of the partial removal of oxidized carbon species by etching (Supplementary Fig. 12d).

We also investigated the effect of the same etching procedure on the composition of unprocessed *dh*-BN, as shown in Supplementary Fig. 11 (upper spectra). The etching step resulted in the removal of saturated defects (B–O and N–C) and a downshift of the *h*-BN characteristic peaks (to 190 and 397.5 eV in the B 1*s* and N 1*s* spectra, respectively). This effect is most probably due to the emergence of defects relative to the creation of $B–N_2$ (or $N–B_2$) sites [34], according to the above-mentioned bombardment-induced ligancy decrease. The B 1*s* peak at 190 eV is also consistent with B–C bonds, which can originate from sputtered products of the etched microrod tips [22,23]. The latter interpretation is further supported by the emergence of a low-binding energy (284.2 eV) component in the C 1*s* spectrum (Supplementary Fig. 11c).

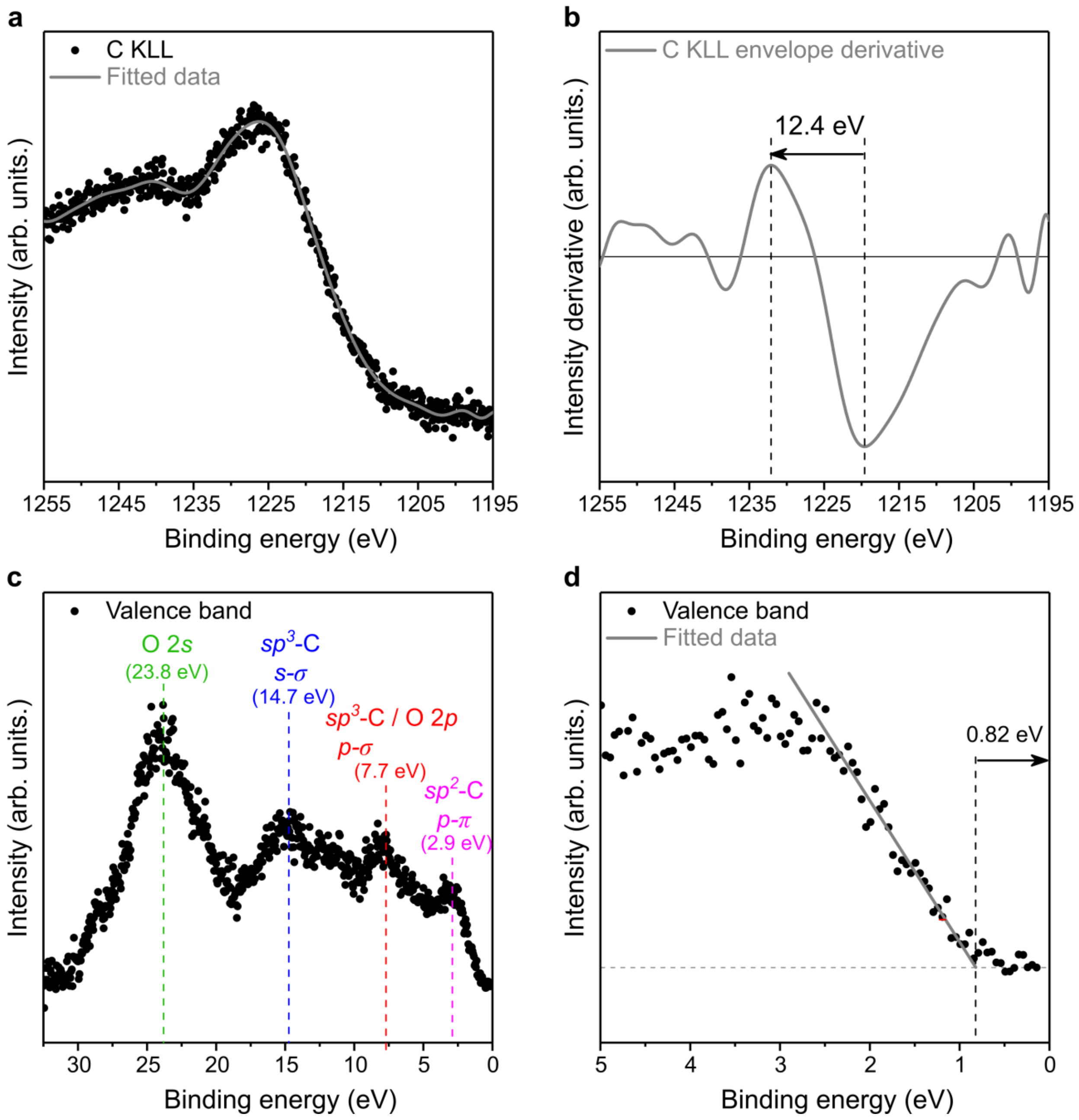


**Supplementary Fig. 13: Carbon Auger and valence band measurements on the carbon microstructure tips. a** HR C KLL Auger transition (symbols) and corresponding envelope generated using polynomial regression (gray line). **b** First derivative of the C KLL Auger envelope displaying the D-parameter of the dominant *sp*$^3$-hybridized carbon at the microstructure tips. **c** XPS valence band measured on the microrod tips of the as-grown microstructure on the quartz substrate. **d** Extrapolated edge (gray line) of the valence band signal (symbols) measured on the tips of as-grown microrods. Source data are provided as a Source Data file.

Next, long (1 mm) carbon microrods were patterned via segmented growth and then positioned on a silicon substrate. The top and bottom lateral regions (toward microrod tips and roots, respectively) of these microrods were examined using XPS (Supplementary Fig. 14a). The XPS measurements yielded similar characteristics throughout the microrods' length, as shown in the survey spectra (Supplementary Fig. 14b). Besides the B, N and C characteristic peaks, one can distinguish the signature of the oxidized silicon substrate. These features correspond to Si 2*s* (~151 eV) and Si 2*p* (~99.7 eV) core-level lines as well as satellite lines exhibiting a regular shift (~17.5 eV), which is characteristic of inelastic energy losses of photoelectrons in silicon (plasmon lines) [13]. The absence of the N 1*s* core level (~398 eV) peak was noted in the survey and HR XPS scans of the N 1*s* core level (Supplementary Fig. 15b). In both cases, the signals were indiscernible from the background noise. While the B 1*s* core level signal (~189.4 eV) was not observed in the XPS survey spectrum, the narrow scans of the B 1*s* core-level region revealed a major feature located at about 185.7 eV and a weak peak at a higher binding energy (Supplementary Fig. 15a). The peak at 185.7 eV stems from the plasmon loss of silicon, as explained above [35,36], while the weak peak (~190 eV) could be intuitively assigned to B−C bonds given the absence of nitrogen and the higher binding energies for boron bonded to oxygen [16,17,22]. Although the XPS narrow-scans of the B 1*s* core level, recorded at different regions of the top and bottom parts of the microrods, yielded noisy signals with scattered positions for this weak component (~190 eV), they reveal insignificant contents of boron with respect to carbon. In addition, the C 1*s* core-level analysis (Supplementary Fig. 15c) excludes the formation of C−B bonds expected at lower binding energies (in the 282–284 eV range) with respect to graphitic carbon [16,23,37]. Indeed, the C 1*s* narrow scan shows a prominent feature at 284.8 eV corresponding to graphitic carbon (C−C bonds), and a smaller peak at 286.55 eV attributed to oxidized carbon (C−O bonds). In agreement with Raman analyses (Fig. 2a and Supplementary Table 1), this result further indicates that the surface of microrods' shells is composed of graphitic carbon domains, as well as defected carbon sites that are passivated by oxygen upon exposure to air, either by oxidation in graphitic domains (C−O−C bonds) or by hydroxylation of surface carbon defects (C−OH bonds). One should keep in mind that the XPS analysis performed rendered only the chemical environments of the microrods' shell surfaces. The latter was found to be predominantly

composed of graphitic carbon, for which the corresponding peak (*i.e.*, the C−C feature) is better resolved on the bottom than on the microrod top (Supplementary Fig. 15c), which supports the growth model described in Fig. 3g. Thus, we infer that the light-induced excitation of *dh*-BN defected sites triggers the photocatalytic decomposition of the hydrocarbon molecules (from the reactant gas) and the formation of graphitic layers that are consequently more structured at the bottom of microrods than at the top, since the microrod tips containing defected carbon with *dh*-BN clusters constitutes the growth front edges. Finally, one can notice that the shake-up satellites relative to $\pi$-$\pi^*$ transitions (~291 eV) cannot be resolved in the C 1*s* HR spectrum, probably due to their weak intensity with respect to the graphitic carbon peak.

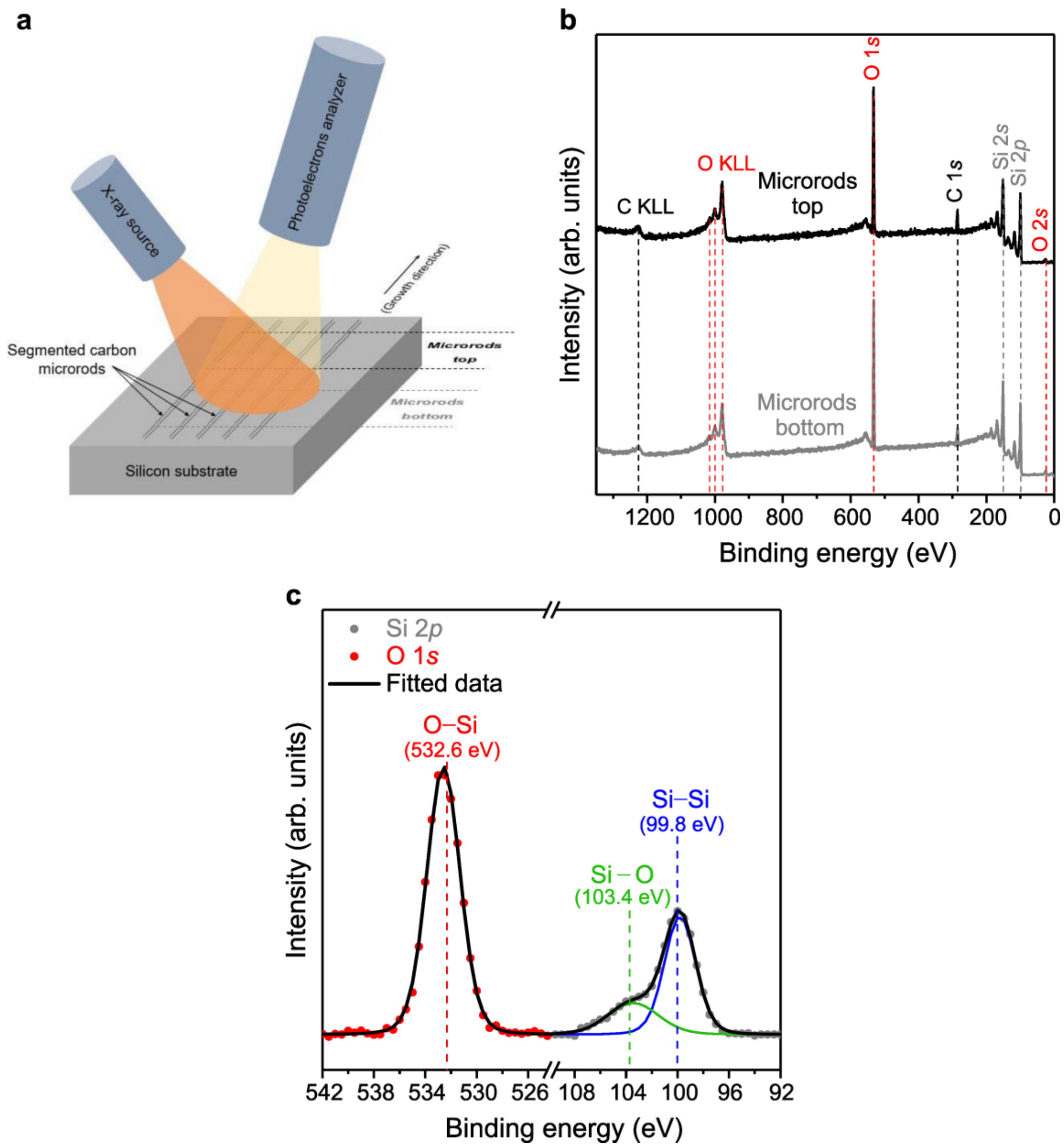


**Supplementary Fig. 14: Experimental setup and XPS measurements on the carbon microrod side. a** Schematic of the XPS characterization method of 1 mm-length carbon microrods obtained using segmented growth and then placed on a supporting silicon substrate. The scheme illustrates the so-called "microrods top" and "microrods bottom" parts mentioned in the text. **b** XPS survey spectra recorded on the top and bottom parts of the 1-mm-length carbon microrods. **c** XPS scans of the Si 2*p* and O 1*s* photoelectron lines used to carry out the quantitative analysis of Si and O atomic concentrations. The core-level signals are fitted with sums (black lines) of Gaussian-Lorentzian functions (colored lines). The Si 2*p* signal consists of contributions from the bulk substrate (Si−Si bonds) and the native oxide (Si−O bonds). Source data are provided as a Source Data file.

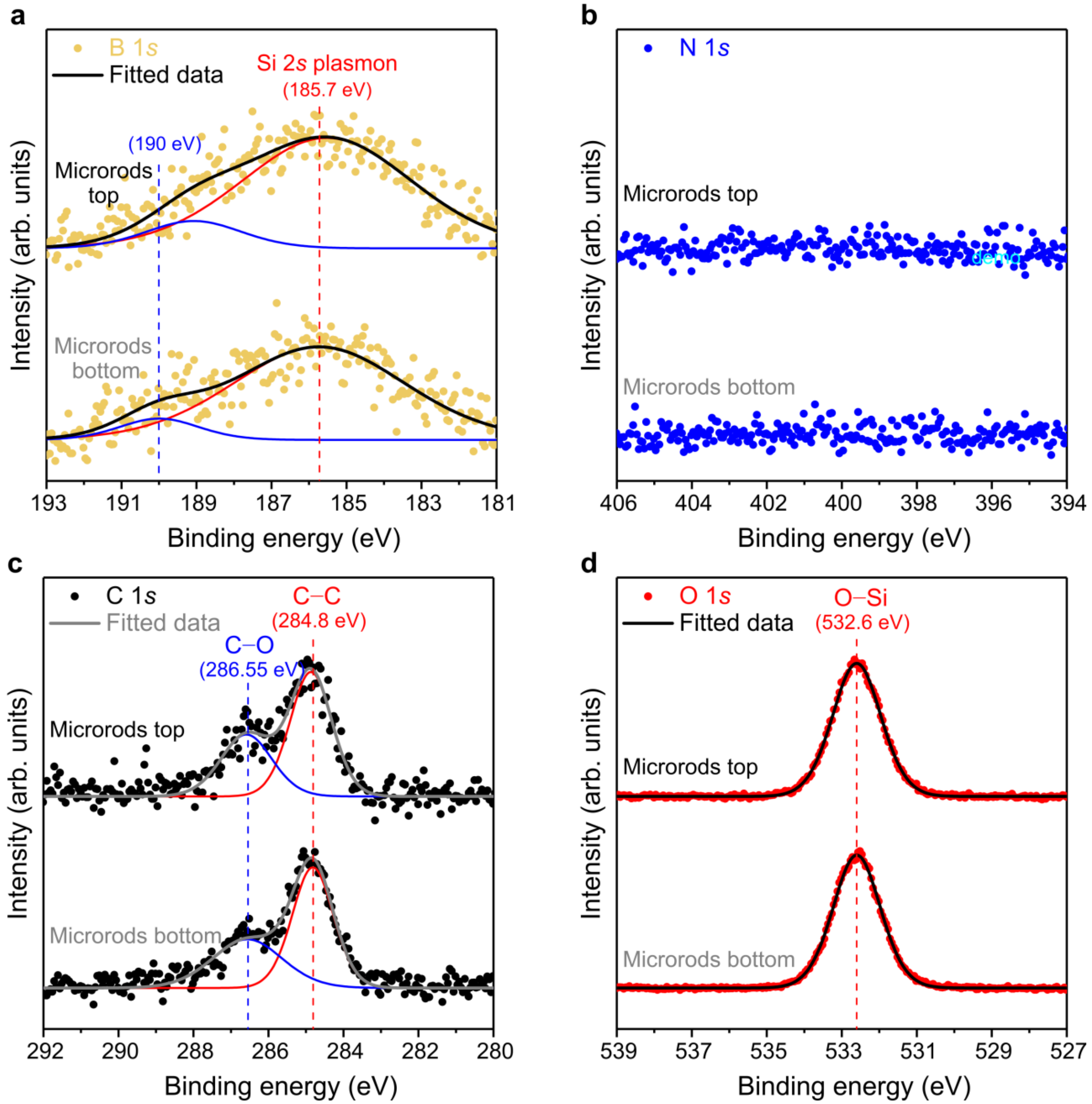


**Supplementary Fig. 15: HR XPS analysis of the carbon microrod side. a–d** XPS narrow scans (symbols) of B 1*s* (**a**), N 1*s* (**b**), C 1*s* (**c**), and O 1*s* (**d**) lines fitted with sums (black/gray lines) of prominent Gaussian-Lorentzian functions (colored lines) for the top and bottom lateral parts of carbon microrods on a natively oxidized silicon substrate. Source data are provided as a Source Data file.

Taking into account the probing depth sampled by XPS (few nanometers), photoelectrons from the substrate originate from two distinct environments relative to

bulk silicon and native oxide. Using the intense Si 2*p* signal of the survey spectrum (rather than that of Si 2*s* photoelectrons, which overlaps with the B 1*s* core-level region), we investigated the contributions (*i.e.*, the atomic amounts) of elemental silicon (~99.8 eV) and silicon bonded to oxygen (~103.4 eV). The collected O 1*s* photoelectrons signal (~532.6 eV) from the same survey spectrum was also analyzed concurrently (Supplementary Fig. 14c). Accordingly, atomic concentrations are 43% for elemental silicon, 18.5% for silicon bonded to oxygen, and 38.5% for oxygen, indicating the quite stoichiometric composition of the native oxide (*i.e.*, a Si:O atomic concentration close to 1:2). Consistently, the O 1*s* HR scan, shown in Supplementary Fig. 15d, exhibits a single and relatively sharp (FWHM 1.5 eV) feature at 532.6 eV corresponding to the substrate oxide. As a result, the O 1*s* analysis shows that the detected oxygen primarily comes from silicon oxide, while it does not definitively rule out the occurrence of other environments, such as carbon oxidized states.

**Creation of multi-segmented and multi-branched carbon microstructures**

Carbon growth was monitored in two ways, first by monitoring the luminescence emission of the reacting structure with the camera (Supplementary Movie 1) or by analyzing the luminescence emission using the CCD detector of the Raman/photoluminescence measurement (Supplementary Figs. 8 and 9).

Carbon microrods with high aspect ratio, up to ~500, were obtained using two different approaches. The first approach consists of a segmented growth: the laser is first focused on the surface of the catalyst and maintained at this position for 30 s, before refocusing the laser at the microrod tip. Successive refocusing of the laser led to the formation of up to 2 mm long rods, as shown in Supplementary Fig. 16. The structures obtained with this approach exhibit a segmented morphology corresponding to the successive 30 s sequences of carbon growth.

The second approach to obtain long microrods is to move the laser focus continuously during growth. The laser focus displacement can either be adapted during the growth (faster at the start (~50 μm/s) and slower toward the end (< 2 μm/s)) or fixed at a slow rate during the entire process (< 2 μm/s). Carbon microstructures more than 2 mm long

were obtained with this approach. The structures exhibit a constant diameter during this process (Supplementary Fig. 17).

Multi-branched structures were obtained by varying the angle of incident light. In Fig. 3e, the Y-shaped microstructure was obtained by changing the angle of the reactor with respect to the laser illumination and focusing the laser on the tip of the first segment of the microrod (*i.e.*, according to the segmented growth approach). The second branch was obtained by changing the angle a second time and refocusing the laser on the tip of the first segment. To produce the micro-antenna structure (Fig. 3f), we first create a long microrod as described above. We changed the orientation of the rod to refocus the laser light on the shell of the microrod. The 2$^{nd}$ rod was grown by displacing the laser in the Z direction during growth. The structure was then rotated by manipulating the reactor a second time. The structures obtained on the 3$^{rd}$ plane (*i.e.*, the structures parallel to the 3$^{rd}$ rod) were produced with the same approach consisting in displacing the laser focus during growth. Multiple rods were obtained by moving the laser focus along the axis of the 1$^{st}$ rod.

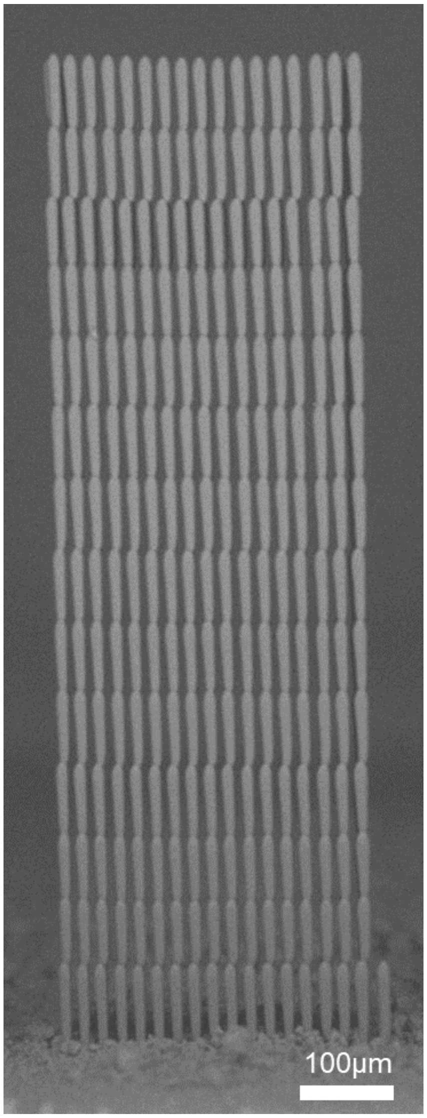


**Supplementary Fig. 16: SEM image of 1 mm-long segmented carbon microstructures.** The segmented growth was performed by refocusing the laser at the tip of the microstructures after every 30 s growth. The rods were obtained with an illumination of 532 nm using a 10× objective and laser power of 100 mW.

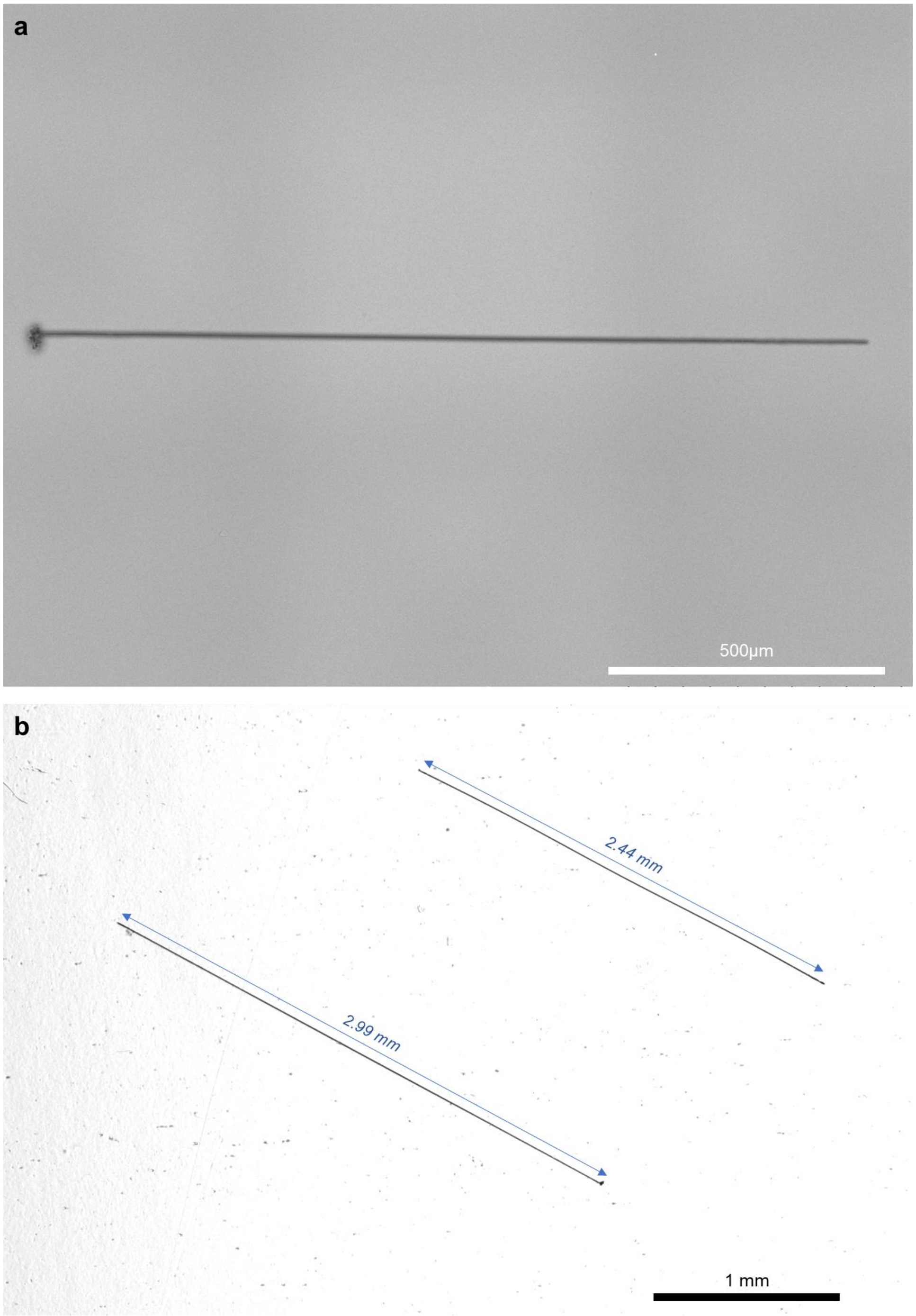


**Supplementary Fig. 17: Images of long carbon microstructures. a** SEM image of a 1.5 mm-long carbon rod obtained by continuously moving the focal point of the laser to drive growth with an illumination of 532 nm using a 10× objective and laser power of 10 mW. The speed of motion was set at 5 μm/s for this rod. **b** Optical image of 2.99 mm and 2.4 mm-long carbon rods obtained by continuously moving the focal point of the laser to drive growth with an illumination of 532 nm using a 10× objective and laser power of 10 mW. The speed of motion was set at 5 μm/s for these rods.

## Evaluation of the carbon microrod-based devices

### Electrical properties of carbon microrods

The I-V curves and Nyquist plot of the structures indicate that the long rods (>1 mm) are simple resistors. The resistance of the structures is about 10 times higher than that of commercial carbon fibers, as shown in Supplementary Fig. 18.

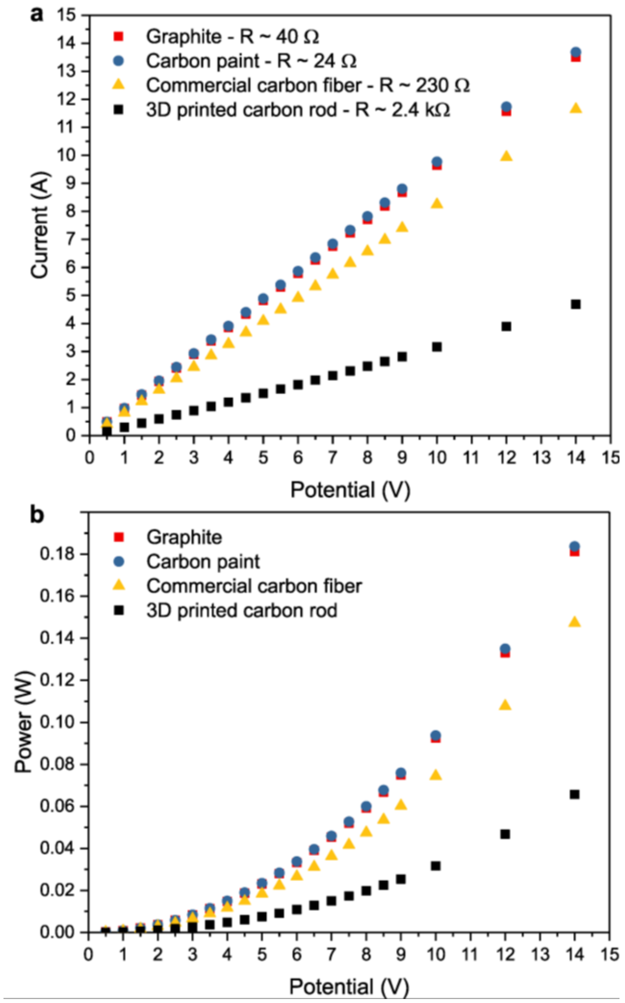


**Supplementary Fig. 18: Comparison of the electrical properties of the 3D printed carbon structures, graphite, carbon paint, and commercial carbon fibers. a**,**b**, I-V characteristics (**a**) and power curves (**b**) of the different measured carbon materials. Source data are provided as a Source Data file.

## Strain sensing properties of carbon microrods

We investigated the carbon microrods response to mechanical strain. A single carbon microrod was adhered across electroless nickel immersion gold (ENIG)-coated test pads, spaced of 0.7 mm, on a flexible polyimide substrate. The mounted assembly was affixed to a polyester hinge in a small vise. Positive curvature was induced by moving the jaws of the vise closer together while pushing on the end of the polyester support (Supplementary Fig. 19). The change in resistance was monitored in a quarter Wheatstone bridge configuration with a multiturn potentiometer as a balancing resistor. A 5 V DC excitation voltage was applied, and the potentiometer was adjusted until the bridge potential was about 0 V. The potential across the bridge was measured in timed intervals while flexing the carbon microrod. Data was acquired using National Instrument Labview interfaced to a SCXI-1600 16-bit digitizer and a SCXI-1520 8-Channel Strain/Bridge Module equipped with a SCXI-1314 Universal Strain Terminal Block.

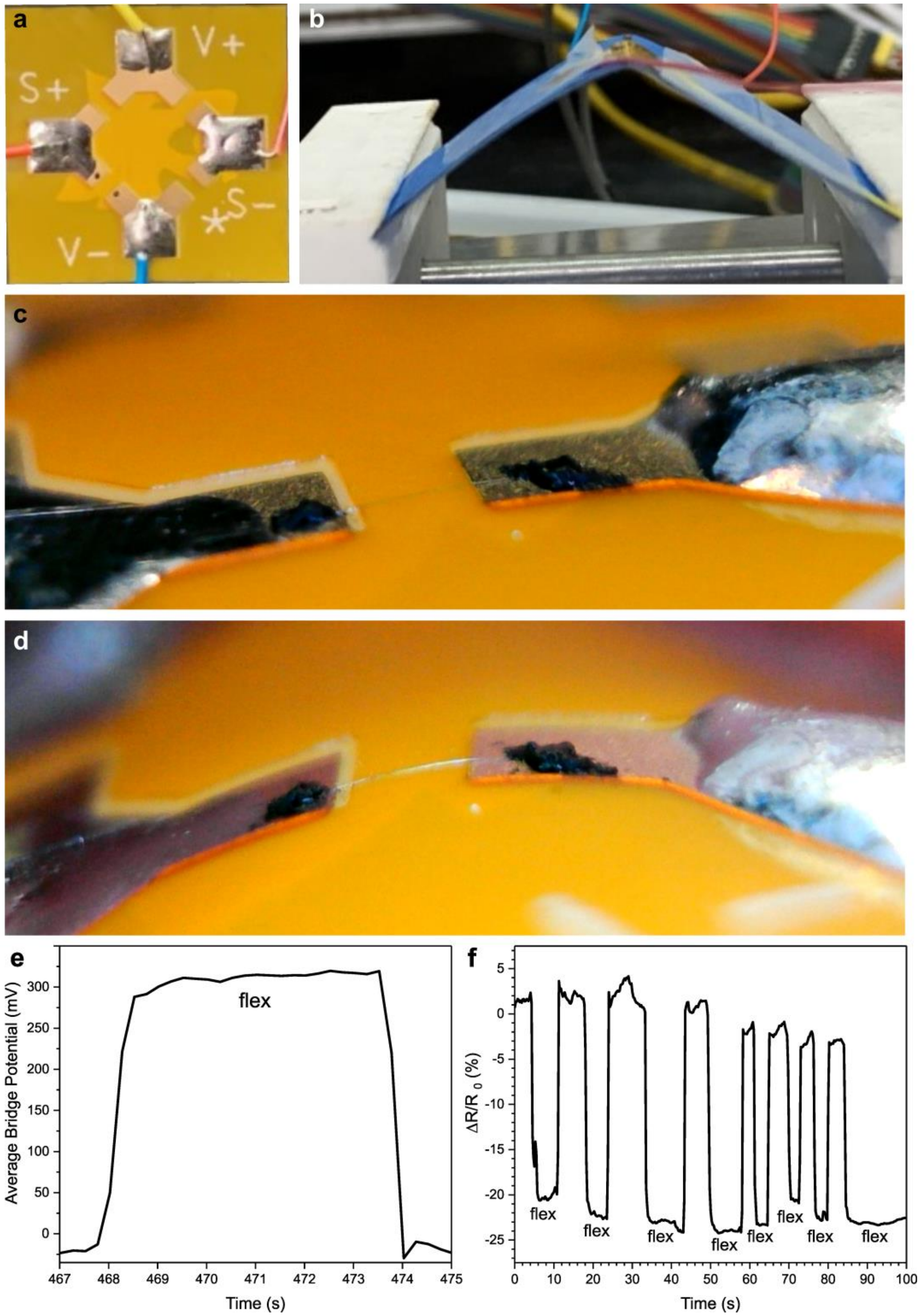


**Supplementary Fig. 19: Single carbon microrod-based strain sensor. a** Wheatstone bridge on a flexible polyimide substrate. **b** Photograph of the flexed system used to measure the effect of strain on the resistance of the carbon microrod. **c**, **d** Photographs of the carbon microrod on the device before (**c**) and during flexing (**d**). **e** Effect of flexing the device on the bridge potential. **f** Effect of successive flexing cycles on the fractional change in the device electrical resistance. Source data are provided as a Source Data file.

## Temperature sensing properties of carbon microrods

Similarly, we examined the properties of carbon microrods in temperature sensing. In these measurements, a single carbon microrod was adhered across hot air solder leveling (HASL)-coated test pads spaced of 0.7 mm on a FR-4 substrate. The mounted assembly was placed in a custom optical cell with 4 electrical feedthroughs. Two were used to sense the fiber resistance and two were used for a PT100 RTD positioned underneath the test board. The temperature was controlled via optical heating through a sapphire window. The change in resistance was monitored in a quarter Wheatstone bridge configuration with a multiturn potentiometer as a balancing resistor (Supplementary Fig. 20). An excitation voltage of 5 V DC was applied, and the potentiometer was adjusted until the bridge potential was near 0. The potential across the bridge was measured in timed intervals heating the fiber. Data was acquired using National Instrument Labview interfaced to a SCXI-1600 16-bit digitizer and a SCXI-1520 8-Channel Strain/Bridge Module equipped with a SCXI-1314 Universal Strain Terminal Block. The temperature was monitored via a PT100 thermocouple positioned under the test board. The RTD signal was amplified by a MAX31865 Temperature Sensor Amplifier and polled via USB.

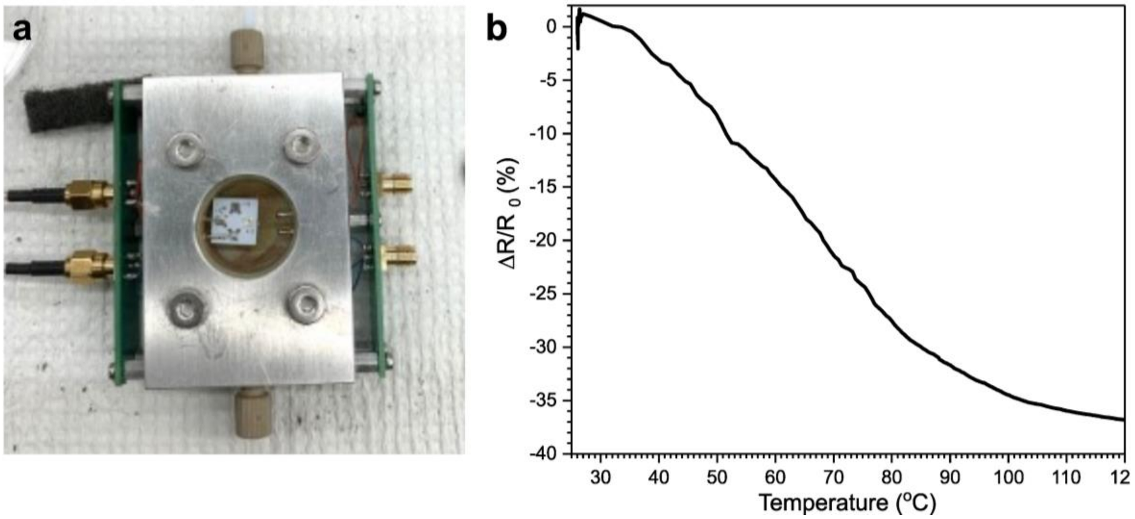


**Supplementary Fig. 20: Single carbon microrod-based temperature sensor. a** Picture of the Wheatstone bridge setup used for temperature sensing measurements. **b** Fractional change in electrical resistance as a function of the device temperature. Source data are provided as a Source Data file.

## REFERENCES


1 Ding, Y. *et al.* Defect engineering in boron nitride for catalysis. *MRS Communications* **8**, 1236-1243, doi:10.1557/mrc.2018.113 (2018).

2 Sadezky, A., Muckenhuber, H., Grothe, H., Niessner, R. & Pöschl, U. Raman microspectroscopy of soot and related carbonaceous materials: Spectral analysis and structural information. *Carbon* **43**, 1731-1742, doi:https://doi.org/10.1016/j.carbon.2005.02.018 (2005).

3 Seong, H. J. & Boehman, A. L. Evaluation of Raman Parameters Using Visible Raman Microscopy for Soot Oxidative Reactivity. *Energy & Fuels* **27**, 1613-1624, doi:10.1021/ef301520y (2013).

4 Cuesta, A., Dhamelincourt, P., Laureyns, J., Martínez-Alonso, A. & Tascón, J. M. D. Raman microprobe studies on carbon materials. *Carbon* **32**, 1523-1532, doi:https://doi.org/10.1016/0008-6223(94)90148-1 (1994).

5 Dresselhaus, M. S. & Dresselhaus, G. Intercalation compounds of graphite. *Advances in Physics* **30**, 139-326, doi:10.1080/00018738100101367 (1981).

6 Wang, S., Xing, X., Zhang, X., Wang, X. & Jing, X. Room-temperature fully recyclable carbon fibre reinforced phenolic composites through dynamic covalent boronic ester bonds. *Journal of Materials Chemistry A* **6**, 10868-10878, doi:10.1039/C8TA01801D (2018).

7 Sze, S. K., Siddique, N., Sloan, J. J. & Escribano, R. Raman spectroscopic characterization of carbonaceous aerosols. *Atmospheric Environment* **35**, 561-568, doi:10.1016/S1352-2310(00)00325-3 (2001).

8 Dippel, B., Jander, H. & Heintzenberg, J. NIR FT Raman spectroscopic study of flame soot. *Physical Chemistry Chemical Physics* **1**, 4707-4712, doi:10.1039/A904529E (1999).

9 Dippel, B. & Heintzenberg, J. Soot characterization in atmospheric particles from different sources by NIR FT Raman spectroscopy. *Journal of Aerosol Science* **30**, S907-S908, doi:doi.org/10.1016/S0021-8502(99)80464-9 (1999).

10 Xiong, J., Di, J., Xia, J., Zhu, W. & Li, H. Surface Defect Engineering in 2D Nanomaterials for Photocatalysis. *Advanced Functional Materials* **28**, 1801983, doi:10.1002/adfm.201801983 (2018).

11 Jawhari, T., Roid, A. & Casado, J. Raman spectroscopic characterization of some commercially available carbon black materials. *Carbon* **33**, 1561-1565, doi:https://doi.org/10.1016/0008-6223(95)00117-V (1995).

12 Grant, J. T. *et al.* Selective oxidative dehydrogenation of propane to propene using boron nitride catalysts. *Science* **354**, 1570-1573, doi:10.1126/science.aaf7885 (2016).

13 Chastain, J. & King Jr, R. C. Handbook of X-ray photoelectron spectroscopy. *Perkin-Elmer Corporation* **40**, 221 (1992).

14 Li, J., Li, J., Yin, Y., Chen, Y. & Bi, X. Water-assisted chemical vapor deposition synthesis of boron nitride nanotubes and their photoluminescence property. *Nanotechnology* **24**, 365605, doi:10.1088/0957-4484/24/36/365605 (2013).

15 Gómez-Aleixandre, C., Essafti, A. & Albella, J. M. Kinetic Study of the Diborane/Methylamine Reaction:  Composition and Structure of C−B−N Films. *The Journal of Physical Chemistry B* **104**, 4397-4402, doi:10.1021/jp9929723 (2000).

16 Pakdel, A. *et al.* Facile synthesis of vertically aligned hexagonal boron nitride nanosheets hybridized with graphitic domains. *Journal of Materials Chemistry* **22**, 4818-4824, doi:10.1039/C2JM15109J (2012).

17 Cao, Y. *et al.* B–O Bonds in Ultrathin Boron Nitride Nanosheets to Promote Photocatalytic Carbon Dioxide Conversion. *ACS Applied Materials & Interfaces* **12**, 9935-9943, doi:10.1021/acsami.9b21157 (2020).

18 Puyoo, G., Teyssandier, F., Pailler, R., Labrugère, C. & Chollon, G. Boron carbonitride coatings synthesized by LPCVD, structure and properties. *Carbon* **122**, 19-46, doi:https://doi.org/10.1016/j.carbon.2017.06.024 (2017).

19 Genisel, M. F. *et al.* Bias in bonding behavior among boron, carbon, and nitrogen atoms in ion implanted a-BN, a-BC, and diamond like carbon films. *Journal of Applied Physics* **110**, 074906, doi:10.1063/1.3638129 (2011).

20 Sivaprakash, K., Induja, M., Gomathipriya, P., Karthikeyan, S. & Umabharathi, S. T. Single-step synthesis of efficient nanometric boron carbon nitride semiconductor for photocatalysis. *Materials Research Bulletin* **134**, 111106, doi:https://doi.org/10.1016/j.materresbull.2020.111106 (2021).

21 Torres-Davila, F. E., Molinari, M., Blair, R. G., Rochdi, N. & Tetard, L. Enhancing Infrared Light–Matter Interaction for Deterministic and Tunable Nanomachining of Hexagonal Boron Nitride. *Nano Letters* **22**, 8196-8202, doi:10.1021/acs.nanolett.2c02841 (2022).

22 Paul, R., Voevodin, A. A., Zemlyanov, D., Roy, A. K. & Fisher, T. S. Microwave-Assisted Surface Synthesis of a Boron–Carbon–Nitrogen Foam and its Desorption Enthalpy. *Advanced Functional Materials* **22**, 3682-3690, doi:https://doi.org/10.1002/adfm.201200325 (2012).

23 Jacques, S. *et al.* LPCVD and characterization of boron-containing pyrocarbon materials. *Carbon* **34**, 1135-1143, doi:https://doi.org/10.1016/0008-6223(96)00075-9 (1996).

24 Zhou, H. *et al.* High thermal conductivity of suspended few-layer hexagonal boron nitride sheets. *Nano Research* **7**, 1232-1240, doi:10.1007/s12274-014-0486-z (2014).

25 Ayiania, M. *et al.* Deconvoluting the XPS spectra for nitrogen-doped chars: An analysis from first principles. *Carbon* **162**, 528-544, doi:https://doi.org/10.1016/j.carbon.2020.02.065 (2020).

26 Smith, M., Scudiero, L., Espinal, J., McEwen, J.-S. & Garcia-Perez, M. Improving the deconvolution and interpretation of XPS spectra from chars by ab initio calculations. *Carbon* **110**, 155-171, doi:https://doi.org/10.1016/j.carbon.2016.09.012 (2016).

27 Kim, S. *et al.* A coaxial structure of multiwall carbon nanotubes on vertically aligned Si nanorods and its intrinsic characteristics. *Journal of Materials Chemistry C* **2**, 6985-6990, doi:10.1039/C4TC01251H (2014).

28 Lascovich, J. C. & Scaglione, S. Comparison among XAES, PELS and XPS techniques for evaluation of Sp2 percentage in a-C:H. *Applied Surface Science* **78**, 17-23, doi:https://doi.org/10.1016/0169-4332(94)90026-4 (1994).

29 Lesiak, B. *et al.* C sp2/sp3 hybridisations in carbon nanomaterials – XPS and (X)AES study. *Applied Surface Science* **452**, 223-231, doi:https://doi.org/10.1016/j.apsusc.2018.04.269 (2018).

30 Reinke, P. & Oelhafen, P. Electronic properties of diamond/nondiamond carbon heterostructures. *Physical Review B* **60**, 15772-15781, doi:10.1103/PhysRevB.60.15772 (1999).

31 Endo, K. *et al.* Analysis of Electron Spectra of Carbon Allotropes (Diamond, Graphite, Fullerene) by Density Functional Theory Calculations Using the Model Molecules. *The Journal of Physical Chemistry A* **107**, 9403-9408, doi:10.1021/jp0345710 (2003).
32 Saugnac, F., Teyssandier, F. & Marchand, A. Characterization of C─B─N Solid Solutions Deposited from a Gaseous Phase between 900° and 1050°C. *Journal of the American Ceramic Society* **75**, 161-169, doi:https://doi.org/10.1111/j.1151-2916.1992.tb05459.x (1992).
33 Ottaviani, B. *et al.* Boronated carbons: structural characterization and low temperature physical properties of disordered solids. *Journal of Materials Chemistry* **8**, 197-203, doi:10.1039/A703625F (1998).
34 Bachmann, P. *et al.* A HR-XPS study of the formation of h-BN on Ni(111) from the two precursors, ammonia borane and borazine. *The Journal of Chemical Physics* **149**, 164709, doi:10.1063/1.5051595 (2018).
35 Song, C. *et al.* High-conductive nanocrystalline silicon with phosphorous and boron doping. *Applied Surface Science* **257**, 1337-1341, doi:https://doi.org/10.1016/j.apsusc.2010.08.065 (2010).
36 Zhang, X., Wu, A., Shi, S. & Qin, F. Influence of Ar/$H_2$ ratio on the characteristics of boron-doped nc-Si:H films prepared by electron cyclotron resonance plasma-enhanced chemical vapor deposition. *Surface and Coatings Technology* **228**, S412-S415, doi:https://doi.org/10.1016/j.surfcoat.2012.05.056 (2013).
37 Hou, L., Chen, Z., Liu, X., Gao, Y. & Jia, G. X-ray photoelectron spectroscopy study of cubic boron nitride single crystals grown under high pressure and high temperature. *Applied Surface Science* **258**, 3800-3804, doi:https://doi.org/10.1016/j.apsusc.2011.12.032 (2012).
