# Supplementary material for: Room temperature 3D carbon microprinting
Source: Nat Commun. 2024 Mar 29;15:2745. doi: 10.1038/s41467-024-47076-z (PMC10980711; doi:10.1038/s41467-024-47076-z)
Supplement: Supplementary file 3 — Description of Additional Supplementary Files [file 41467_2024_47076_MOESM3_ESM.pdf]

Description of Additional Supplementary Files

File Name: Supplementary Movie 1

Description: Photoluminescence emitted by the carbon rod tip during growth. The signal is used to guide the growth of millimeter-long rods and adjust the speed at which the laser focus moves.
